# clustur: an R package for clustering features using sparse distance matrices

**DOI:** 10.1128/mra.01238-24

**Published:** 2025-01-30

**Authors:** Gregory Johnson, Sarah L. Westcott, Patrick D. Schloss

**Affiliations:** 1 Department of Microbiology & Immunology, University of Michigan 12266 , Ann Arbor, Michigan, USA; Loyola University Chicago, Chicago, Illinois, USA

**Keywords:** bioinformatics, software, hierarchical clustering, operational taxonomic unit, bacterial taxonomy, microbiome

## Abstract

The clustur R package implements the *de novo* clustering algorithms found in the mothur software package for assigning 16S rRNA gene sequences to operational taxonomic units (OTUs). Making these algorithms accessible through the R ecosystem will foster their further development, broader application, and integration within other R packages.

## ANNOUNCEMENT

Taxonomic classification of 16S rRNA gene sequences has been a persistent challenge in microbial ecology studies because reference databases are incomplete (1). As an alternative, operational taxonomic units (OTUs) have been widely used for describing and comparing microbial communities. Although their biological interpretation is controversial, OTUs are typically defined as a group of sequences that are more than 97% similar or less than 3% dissimilar to each other (2). Methods for applying that definition has resulted in a sizable literature. Three general approaches have emerged for assigning sequences to OTUs: *de novo* clustering, closed reference clustering or phylotyping, and open reference clustering (3–9). These methods are available through popular packages, including mothur and QIIME2 (10, 11).

The clustur R package implements the *de novo* clustering algorithms implemented in mothur. The package name references its focus on clustering and the names of its predecessors DOTUR and mothur (10, 12). This package was developed to help address two issues. First, users would be able to more easily integrate the type of analysis that mothur specializes in with popular analysis and visualization packages within the R package ecosystem. Second, by making the code behind mothur’s clustering functions accessible through the R language, we hope to encourage further development of the algorithms behind these functions and analyses based on the output of the functions. The clustur package implements hierarchical clustering algorithms, including the furthest, nearest, unweighted (i.e., average), and weighted neighbor clustering algorithms and the OptiClust algorithm. Functions implementing the hierarchical algorithms already exist within R; however, their implementations within clustur make use of a sparse input distance matrix and output data for a single distance threshold. The benefits of censoring distances larger than the threshold and only outputting data for a single threshold include a smaller memory requirement and faster execution times (4). clustur makes use of the Rcpp R package to implement C ++ code originally written for the mothur software package to preserve the speed of the functions.

Users can install the clustur package via CRAN or through the devtools package’s install_github function. The primary input to clustur’s functions is a sparse distance matrix and a count file. The sparse distance matrix is a data.table package object with two columns indicating the identifiers of the sequences being compared and a column with the distance between those sequences; data for comparisons with a distance larger than the desired threshold (e.g., 0.03) do not need to be included. The count file is a data.table package object indicating the number of times a sequence is found in each sample. The cluster functions output two data.table objects. The first one has two columns indicating the sequences and OTU identifiers. The second displays the abundance of each sequence in each OTU. This has identical functionality to the cluster and make.shared functions from mothur. Detailed vignettes are available within the package to teach users how to install the package, use its functions, and perform downstream analyses, including analysis within the vegan and ggplot2 R packages.

## Data Availability

clustur is available through CRAN, and developmental versions are available through the project’s GitHub website (https://github.com/schlosslab/clustur). The package is available under the GNU General Public License (v3.0).
